# Genomic Epidemiology of Antimalarial Drug Resistance in *Plasmodium falciparum* in Southern China

**DOI:** 10.3389/fcimb.2020.610985

**Published:** 2021-01-08

**Authors:** Fang Huang, Christopher G. Jacob, Shannon Takala-Harrison, Matthew Adams, Heng-Lin Yang, Hui Liu, Zhi-Gui Xia, Shui-Sen Zhou, Lin-Hua Tang, Christopher V. Plowe

**Affiliations:** ^1^National Institute of Parasitic Diseases, Chinese Center for Disease Control and Prevention, Chinese Center for Tropical Diseases Research, Key Laboratory of Parasite and Vector Biology, Ministry of Health, WHO Collaborating Centre for Tropical Diseases, National Center for International Research on Tropical Diseases, Shanghai, China; ^2^Malaria Programme, Wellcome Sanger Institute, Cambridge, United Kingdom; ^3^Center for Vaccine Development and Global Health, University of Maryland School of Medicine, Baltimore, MD, United States; ^4^Malaria Department, Yunnan Institute of Parasitic Diseases, Puer, China; ^5^Duke Global Health Institute, Duke University, Durham, NC, United States

**Keywords:** *Plasmodium falciparum*, antimalarial drugs, artemisinin resistance, microarrays, Southern China

## Abstract

Emerging artemisinin resistance in Southeast Asia poses a significant risk to malaria control and eradication goals, including China’s plan to eliminate malaria nationwide by 2020. *Plasmodium falciparum* was endemic in China, especially in Southern China. Parasites from this region have shown decreased susceptibility to artemisinin and delayed parasite clearance after artemisinin treatment. Understanding the genetic basis of artemisinin resistance and identifying specific genetic loci associated with this phenotype is crucial for surveillance and containment of resistance. In this study, parasites were collected from clinical patients from Yunnan province and Hainan island. The parasites were genotyped using a *P. falciparum*-specific single nucleotide polymorphism (SNP) microarray. The SNP profiles examined included a total of 27 validated and candidate molecular markers of drug resistance. The structure of the parasite population was evaluated by principal component analysis by using the EIGENSOFT program, and ADMIXTURE was used to calculate maximum likelihood estimates for the substructure analysis. Parasites showed a high prevalence of resistance haplotypes of pfdhfr and pfdhps and moderate prevalence of pfcrt. There was no mutation identified on pfmdr1. Candidate SNPs on chromosomes 10, 13, and 14 that were associated with delayed parasite clearance showed a low prevalence of mutants. Parasites from Southern China were clustered and separated from those from Southeast Asia. Parasites from Yunnan province were substructured from parasites from Hainan island. This study provides evidence for a genomic population with drug resistance in Southern China and also illustrates the utility of SNP microarrays for large-scale parasite molecular epidemiology.

## Introduction

Malaria remains one of the most serious infectious diseases in the world. According to the world malaria report, an estimated 228 million cases of malaria worldwide were reported in 2018 compared with 251 million in 2010 and an estimated 405,000 deaths globally in 2018 compared with 585,000 in 2010 (WHO, 2019). *Plasmodium falciparum* has developed resistance to almost all the antimalarial drugs that have been used. More recently, multidrug resistance, including to artemisinin derivatives and partner drugs of *P. falciparum*, has emerged and spread in Southeast Asia (Ashley et al., 2014; Takala-Harrison et al., 2015; Srimuang et al., 2016). The countries of the Greater Mekong Subregion (GMS) are pursuing malaria elimination with an aim to achieve regional malaria elimination by 2030 driven by this emerging multidrug-resistant *P. falciparum* (WHO, 2015). China has launched the National Malaria Elimination Action Plan 2010–2020 with an ultimate goal to interrupt local malaria transmission by 2020. Southern China was historically the main *P. falciparum* endemic region in China, especially Yunnan and Hainan Provinces. Yunnan Province, which shares borders with Myanmar, Vietnam, and Laos, is the key focus of the national malaria elimination program. China was the first country to use artemisinin, and its wide-scale use began in the early 1990s (Tu, 2011). The national malaria treatment policy of China was updated in 2016, and artemisinin-based combinations are first-line drugs used to treat *P. falciparum* malaria, including dihydroartemisinin-piperaquine, artesunate-amodiaquine, and artemisinin-piperaquine (NHC, 2016). However, parasites in Southern China have shown decreased *in vitro* susceptibility to artemisinin by the ring stage assay and delayed parasite clearance after artemisinin treatment (Huang et al., 2012b; Huang et al., 2015; Wang et al., 2015).

Several molecular markers of *P. falciparum* resistance have been identified. Mutations in *pfcrt*, which encodes a protein located on the digestive vacuole membrane, are responsible for chloroquine (CQ) resistance or treatment failure (Picot et al., 2009; Lakshmanan et al., 2014), and the K76T allele in *pfcrt* has been used for the surveillance of clinical CQ resistance (Djimde et al., 2001; Picot et al., 2009). The *pfmdr1* gene encodes the plasmodial homologue of mammalian multidrug resistance transporters linked with *antimalarial drug resistance (*Holmgren et al., 2007*;* Dahlstrom et al., 2009*;* Vinayak et al., 2010*;* Ferreira et al., 2011*)*. *The single nucleotide polymorphisms (SNPs) at codons N86Y, Y184F, S1034C, N1042D, and D1246Y* of multidrug resistance gene 1 (*pfmdr1*) *are shown to be associated with resistance to mefloquine, lumefantrine*, amodiaquine, CQ, and *artemisinin* (Babiker et al., 2001; Humphreys et al., 2007; Somé et al., 2010). Point mutations of the dihydrofolate reductase (*dhfr*) and dihydropteroate synthase (*dhps*) genes, two key enzymes in the folate biosynthesis pathway, mediate resistance to the antifolate drugs sulfadoxine and pyrimethamine (SP), respectively, and have been well described (Gregson and Plowe, 2005). The SNPs have been identified in codons 436, 437, 540, 581, and 613 in the *pfdhps* gene and codons 108, 51, 59, 140, 16, and 164 in the *pfdhfr* gene (Kublin et al., 2002). Mutations in the propeller region of a kelch protein (K13) on *P. falciparum* chromosome 13 (*PF3D7_1343700*) were identified to be associated with artemisinin resistance (Ariey et al., 2014). Understanding the genetic basis of antimalarial drug resistance and identifying specific genetic loci associated with this phenotype are crucial for effective surveillance and containment of resistance.

Microarray-based comparative genomic hybridization, a powerful tool for whole genome analyses and the rapid detection of genomic variation that underlies virulence and disease, provides a robust tool for genome-wide analysis of malaria parasites in diverse settings (Carret et al., 2005). One of the key challenges for microarray analysis is the small amount of genomic DNA obtained from clinical malaria isolates, which is insufficient to be tested in the array. A custom, high-density, NimbleGen microarray covering 33,716 SNPs with high-quality SNPs calls from a wide range of parasite DNA samples, was developed for genome-wide analysis of malaria parasites in different settings (Jacob et al., 2014).

This study addresses the genomic epidemiology of antimalarial drug resistance in *P. falciparum* from the Yunnan and Hainan Provinces in Southern China by using this custom NimbleGen microarray.

## Materials and Methods

### Sample Collection and DNA Extraction

Dried blood spots (DBS) on filter paper (Whatman™ 903, GE Healthcare) were collected from the participants in a therapeutic efficacy study before they received antimalarial drug treatment as well as from the individuals with *P. falciparum* confirmed by microscopy or rapid diagnostic test (RDT) through routine surveillance. Genomic DNA was extracted from the DBS following the manufacturer’s instructions (QIAamp 96 DNA Blood Kit, Valencia, CA). Nested polymerase chain reaction (PCR), amplifying the small-subunit rRNA gene of *Plasmodium* spp. (Snounou et al., 1993) was used to confirm the species prior to being tested on the array.

### Ethical Considerations

The studies with human subjects were reviewed and approved by the institutional review board of the National Institute of Parasitic Diseases, Chinese Center for Disease Control. In addition, the samples collected from the therapeutic efficacy study were also approved by the WHO Western Pacific Regional Office, and the studies were registered as clinical trials at https://www.anzctr.org.au under the numbers ACTRN12610001008011 and ACTRN12610001028099. Written informed consent was obtained from patients or guardians.

### Quantitative PCR and Whole Gene Amplification (WGA)

Quantitative PCR was used to amplify the *P. falciparum* gene encoding the 18s ribosomal subunit for each sample (Kamau et al., 2011). The total reaction volume was 25 μl, including 2 μl of sample DNA along with 10 μM probe, 10 μM of each primer, H_2_O, and TaqMan universal PCR master mix (containing AmpliTaq Gold DNA Polymerase, dNTPs, and dUTP). The sequences for the primers and probe were Forward - 5′-GTAATTGGAATGATAGGAATTTACAAGGT-3′, Reverse - 5′-TCAACTACGAACGTTTTAACTGCAAC-3′, Probe - 5′-FAM GAACGGGAG GTTAACAA MGB-3′. The PCR conditions were 15 min at 95°C, 15 s at 95°C, and 45 cycles for 1 min at 60°C. The standard curve for DNA quantification was generated and run on each plate as well as a no-DNA control. The standard curve was derived from purified parasite DNA (NF54 strain) and quantified using SYBR Green. This DNA was diluted to 3, 1.5, 0.75, 0.375, 0.188, 0.094, and 0.047 ng/μl, and each standard and sample was tested in duplicate with the final quantity expressed as the mean of both values. The samples with original parasite DNA quantities less than 2 ng were amplified using WGA with the Qiagen REPLI-g mini kit, following the manufacturer’s instructions.

### SNP Microarray

Parasites were genotyped using a *P. falciparum* SNP microarray. This is a custom NimbleGen 4.2 million probe designed in multiplex format, which comprises 12 identical arrays on each slide (Jacob et al., 2014). One slide is capable of genotyping 33,716 loci within the *P. falciparum* genome. Dual-color labeling was used, and two samples could be hybridized to a single array, yielding 33,716 SNPs for 24 samples in a single experiment. In addition, several slides could be run simultaneously, which made this approach relatively high throughput and low cost. The SNP profiles were examined for the prevalence of validated and candidate molecular markers of drug resistance.

### DNA Labeling

Parasite DNA was concentrated using vacuum centrifugation to a volume of 30–50 μl and heat denatured with 1 OD of 65% random nonamers labeled with cy3 or cy5 for 10 min at 98°C. Denatured DNA was chilled on ice for 2 min and then incubated for 2 h at 37°C with 50 units of Klenow fragment and a 50× dNTP mixture. The reaction was terminated with 0.5 M ethylenediamine tetraacetic acid (EDTA), and DNA samples were precipitated with 5 M NaCl and iso-propanol. Labeled DNA was washed 2–3 times with 80% ice-cold ethanol to remove unincorporated dye. After removal of ethanol, the samples were rehydrated in water, and cy3 and cy5 labeled samples were combined for multiplexing. Samples were dried in a SpeedVac on medium heat for 30 min.

### Hybridization

Hybridization master mix was prepared with 45.67 µl 2× hybridization buffer, 39.6 µl Denhardt’s solution, 18.27 µl hybridization comp and 1.88 µl alignment oligo. It must be mixed very well before use, and 8 µl of hybridization master mix was added to each labeled DNA sample, vortexed well, and quickly spun. The mixture was heat denatured at 95°C for 5 min and stabilized at 42°C prior to loading onto the array. Loaded samples were hybridized on the NimbleGen hybridization station for 16–24 h at 42°C.

### Slide Washing and Scanning

Slides were disassembled in a dish containing wash buffer 1 at 42°C and then washed in wash buffer 1, wash buffer 2, and wash buffer 3 for 2 min, 1 min, and 15 s, respectively. The slides were washed and subsequently dried in the Slide Washer 12 Array Processing System. Microarrays were scanned with a NimbleGen MS 200 Microarray Scanner at 2 μm using “auto gain” to automatically adjust the scanning parameters on an individual array basis.

### Data Analysis

Spearman’s rank correlation was used to evaluate the relationship of DNA quantity between the original extraction and post-WGA and the relationship between parasitemia and the original DNA quantity by SAS software (SAS Institute Inc., Version 9.2, Cary, NC, USA). A *P* value of <0.05 was used to evaluate the differences with statistical significance. The SNP call rate and SNP call accuracy were calculated based on the intensity of each probe using the heuristic algorithm written in PERL (Jacob et al., 2014) and standard outputs from the Roche NimbleScan (v2.6) software. The samples with the missing calls of the SNPs associated with antimalarial drug resistance are removed in the data analysis. The structure of each parasite population was evaluated by principal component analysis (PCA) using the EIGENSOFT program (Price et al., 2006), and ADMIXTURE (Alexander et al., 2009) was used to calculate maximum likelihood estimates of the most probable number of ancestral populations (K) based on data of the SNPs for the substructure analysis. The SNP data of Southeast Asia samples used in PCA analysis are from the published study (Takala-Harrison et al., 2015).

## Results

### Sample Information

**Figure 1:**
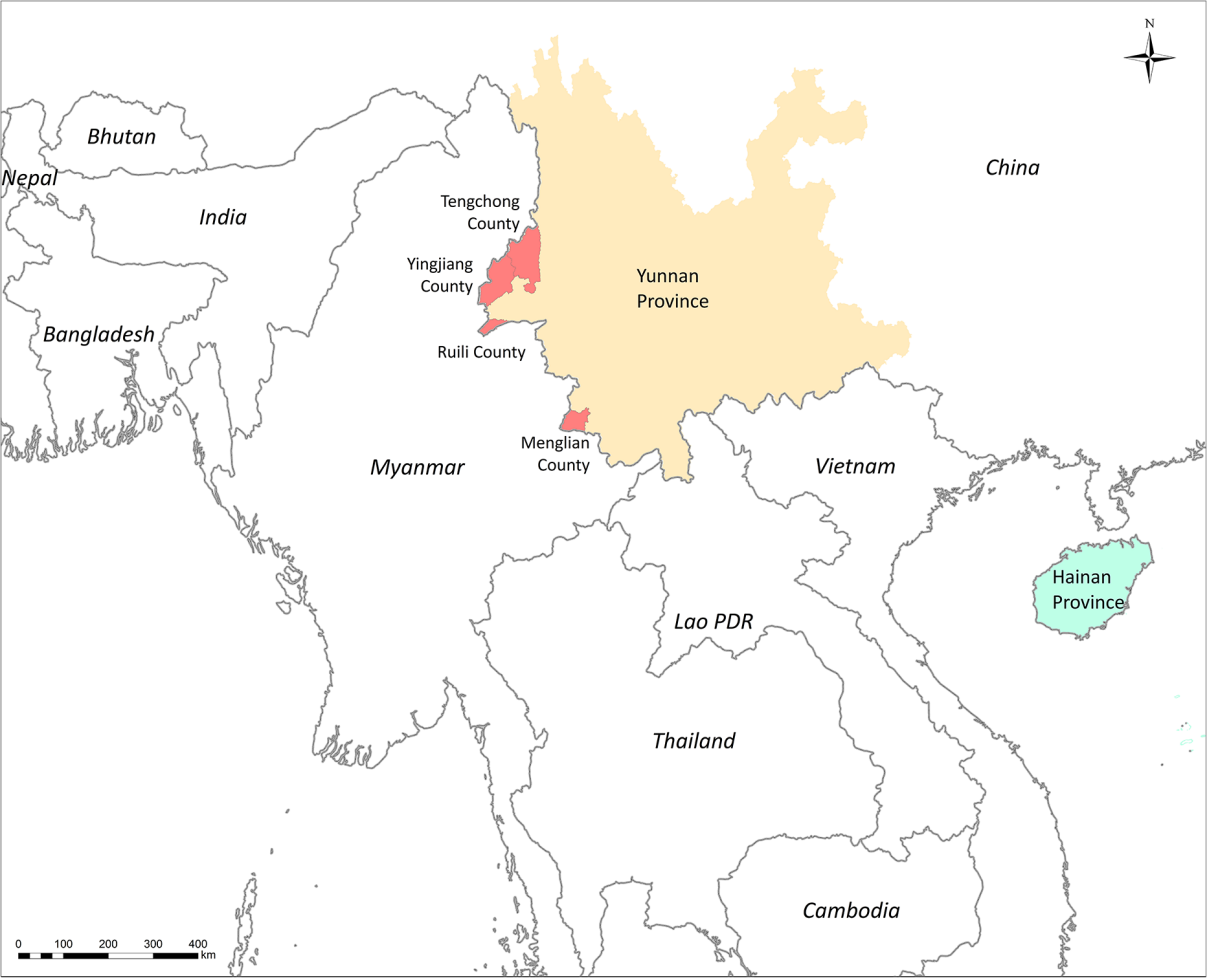
Sites for sample collection in Southern China and countries of samples collection in PCA analysis.

The DBS were collected from *P. falciparum–*infected patients before antimalarial drug treatment. These cases were diagnosed by microscopy or RDT before sample collection. A total of 256 DBS samples were collected from Yingjian, Menglian, and Tengchong in Yunnan Province from 2009 to 2012 (**Figure 1**). Among them, 65 isolates also had parasitemia data from a level 1 microscopist who was certificated by the WHO. Another 11 DBS from Hainan Province collected in 2007 were only used in subpopulation structure analysis (**Table 1**).

**Table 1:** Sites and years of collection of dry blood samples of *P. falciparum* in Southern China.

Study site		Year				Total
	2007	2009	2010	2011	2012	
Yingjiang		84	28		54	166
Ruili	16					16
Menglian		44		11		55
Tengchong					19	19
Hainan	11*					
**Total**		**144**	**28**	**11**	**73**	**256**

The 11 samples from Hainan were only used in the population structure analysis.

### DNA Quantity

**Figure 2:**
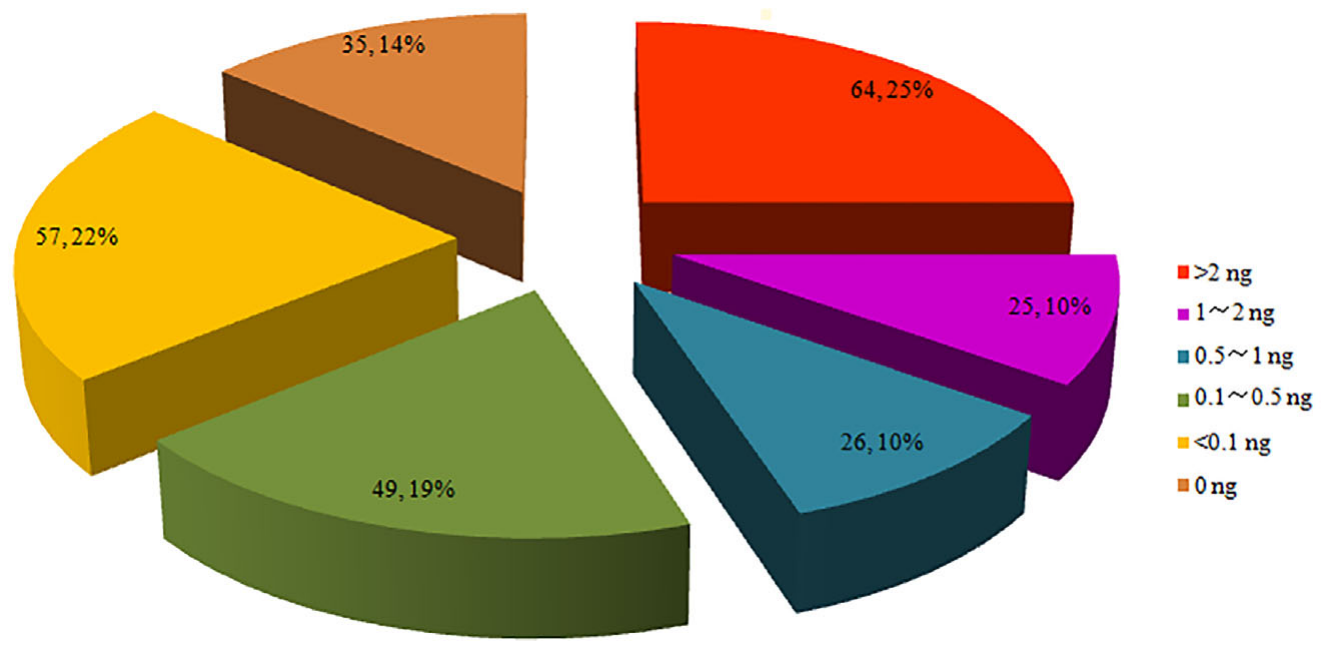
Proportion of parasite genomic DNA evaluated by 18s rRNA qPCR.

After the genomic DNA was extracted, 86.3% (221/256) were successfully quantified of *P. falciparum* genomic DNA, and 35 samples that were tested failed in the quantitative PCR. The threshold for total DNA to be tested in the microarray was 2 ng. The highest DNA quantity in the DBS was 77.51 ng. A total of 64 samples contained more than 2 ng of genomic DNA, which accounted for 25.0%; 25 samples contained DNA between 1 and 2 ng, 75 samples between 0.1 and 1 ng and 57 less than 0.1 ng. The proportion in each range of DNA quantity is shown in **Figure 2**.

**Figure 3:**
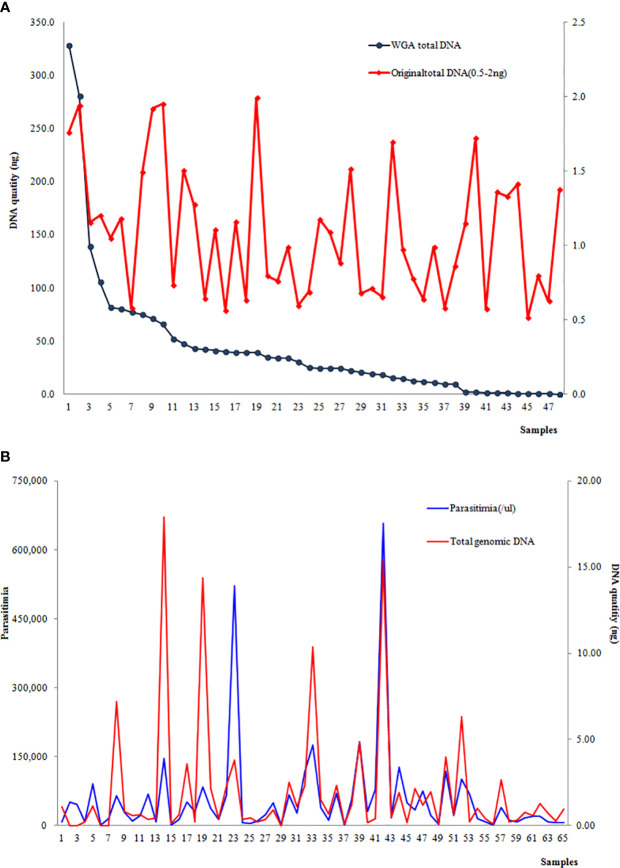
**(A)** Correlation between the DNA concentration of the original extraction and post-WGA; **(B)** Correlation between parasitemia and the original DNA quantity.

At the first step, a total of 48 samples with the original DNA at 0.5–2 ng underwent whole-genome amplification, and 85.6% (43/48) had an increased amount of genomic DNA. The post-WGA DNA amount in 39 samples was more than 2 ng, and the highest post-WGA DNA was 186 times the original DNA amount. Nevertheless, the DNA concentration of post-WGA was not correlated with the original DNA concentration (**Figure 3A**). When the relationship between the parasitemia and DNA quantity was analyzed, there was a significant relation between parasitemia and DNA quantity (*r*=0.6701, *P*<0.001) (**Figure 3B**). Second, another 49 samples with the original DNA < 0.5 ng underwent WGA, and 32 samples were increasing the DNA amount to more than 2 ng. Considering the lower of the original DNA of these 49 samples, the relationship analysis between the original DNA and post-WGA DNA did not include these 49 samples.

### SNP Call Rate

**Figure 4:**
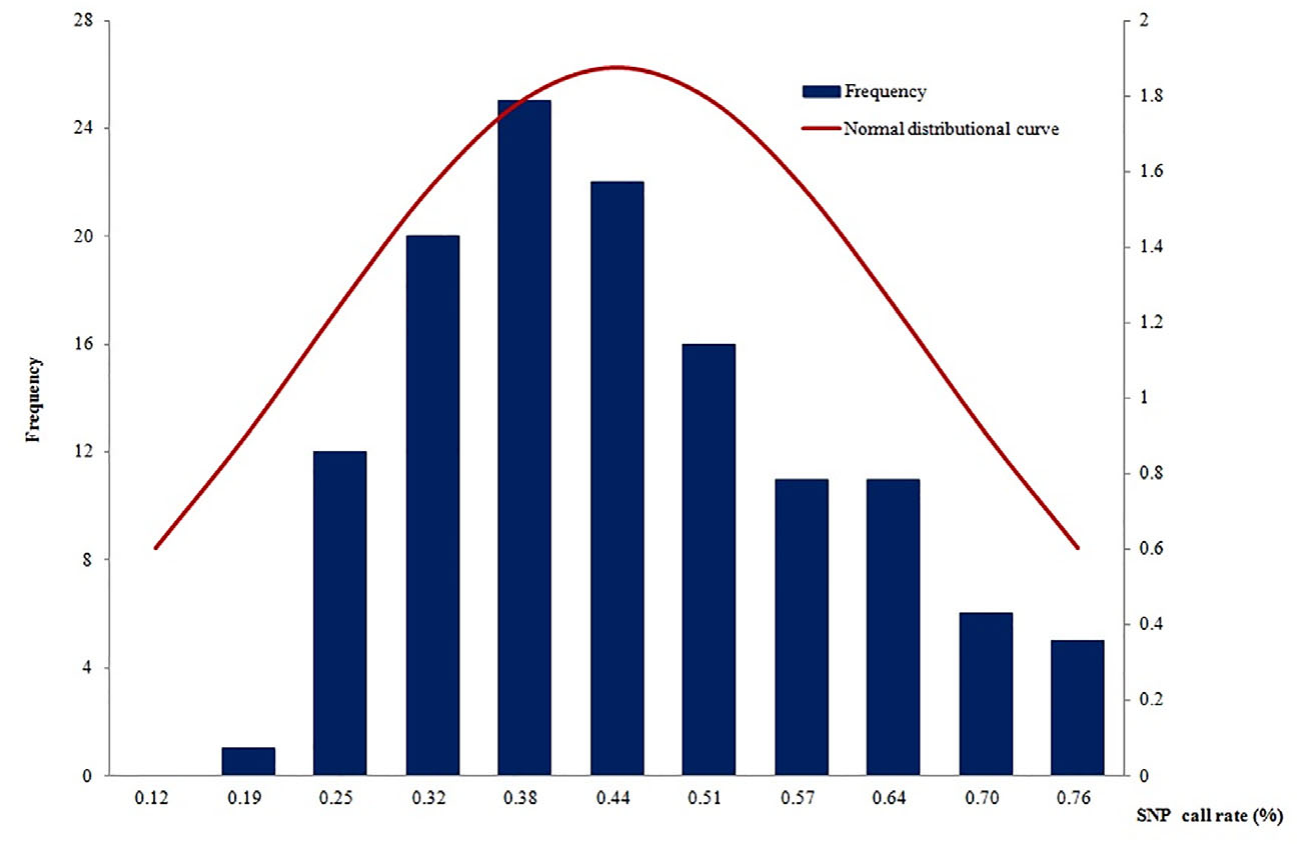
The SNP call rates were not in a normal distribution (mean: 44.38%, standard deviation: 0.17168, *P*<0.01).

A total of 135 samples (including 64 with original DNA >2 ng and 71 with post-WGA DNA>2ng) had an average call rate of 44.4% (**Figure 4**). The highest call rate was 89.1%, and the lowest was 18.5%. The SNP call rates were not in a normal distribution by using the Shapiro-Wilk normality test of SAS software (mean: 44.4%, standard deviation: 0.17168, *P*<0.01). The samples with the highest call rate also had the highest parasite DNA level. In addition, there was a strong correlation between DNA quantity and call rate (data not shown), which demonstrated that DNA quantity was a good predictor of the number of SNPs called.

### Molecular Markers

A total of 27 SNPs related to antimalarial drug resistance were tested through the SNP microarray. These SNPs were from *pfcrt*, *pfmdr1*, *pfdhfr*, *phdhps*, and another four SNPs (MAL10:688956, MAL13:1718319, MAL13:1719976, MAL14:718269) located on chromosomes 10, 13, and 14, which were associated with delayed parasite clearance time. The location of each SNP is shown in the **Supplementary File 1**.

**Figure 5:**
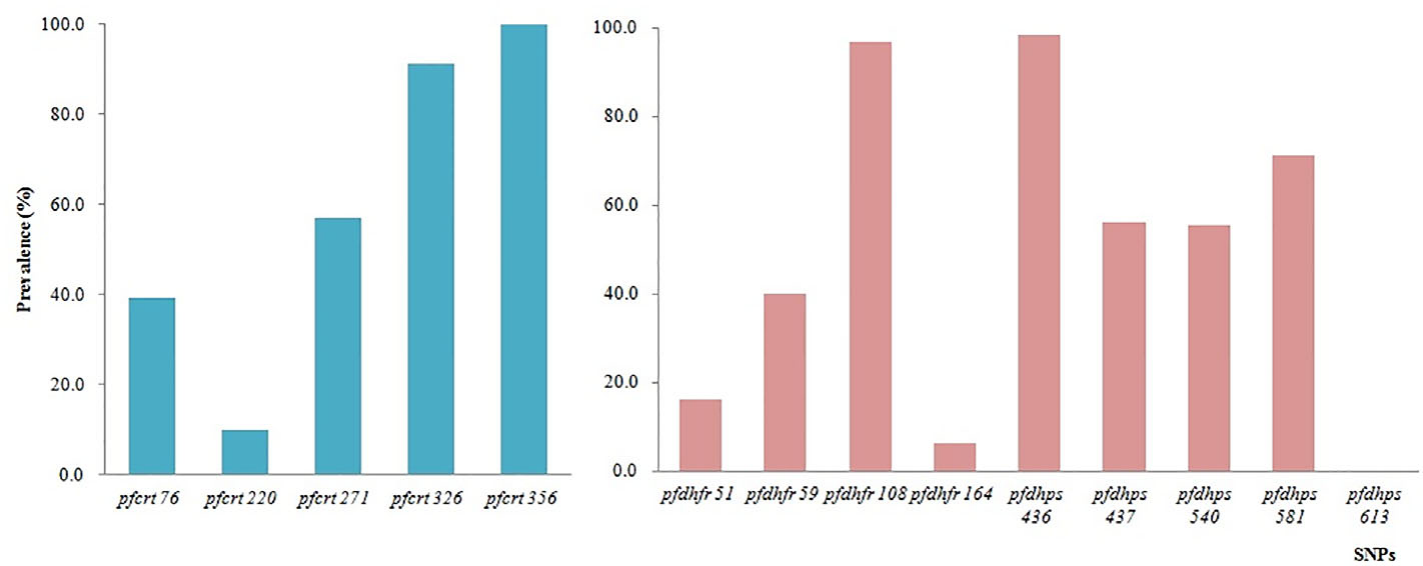
Prevalence of constructed haplotypes of SNPs in *pfcrt*, *pfdhfr*, and *pfdhps* at different codons.

In total, eight codons of *pfcrt* were tested, and no mutations were identified at codons 74, 75, and 371 (**Figure 5**). All 66 samples tested successfully were 100.0% mutant at *pfcrt* 356 (66/66), followed by codons 326 (91.2%, 31/34), 271 (57.1%, 24/42), 76 (39.3%, 11/27), and 220 (9.7%, 3/31). All the SNP mutations involving codons N86Y, Y184F, S1034C, N1042D, and D1246Y of *pfmdr1* were wild type.

A total of 10 SNPs of *pfdhfr* and *pfdhps* associated with antifolate drugs, located on chromosomes 4 and 8, respectively, were tested on the microarray. The highest prevalence of *pfdhfr* was at codon 108 (96.7%, 29/30), followed by codons 59 (40.0%, 16/40), 51 (16.0%, 4/25), and 164 (6.3%, 3/48). No mutations were identified at codon 51. The prevalence of *pfdhps* mutants was consistent with that of *pfdhfr*. The prevalence of *pfdhps* mutation at codons 436, 437, 540, and 581 was 98.5%, 56.3%, 55.6%, and 71.2%, respectively (**Figure 5**).

Candidate SNPs on chromosomes 10, 13, and 14 that were associated with delayed parasite clearance showed low prevalence of mutants. The SNP at MAL13:1719976 showed a 100.0% mutation rate. Nevertheless, the other three had low mutant prevalence or were wild type.

### Population Structure

**Figure 6:**
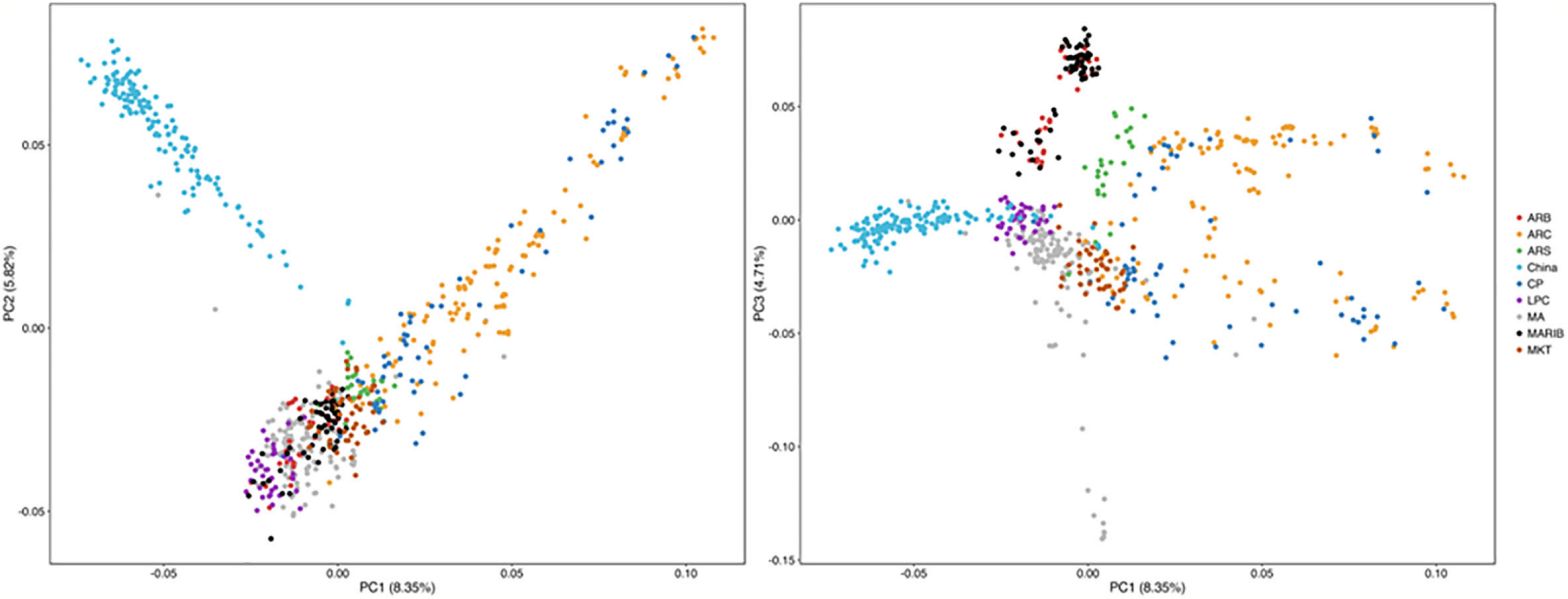
Parasites from Southern China were clustered with and separated from parasites from Southeast Asia by PCA. ARB/MARIB: Bangladesh; ARC/CP: Western Cambodia; ARS: Northwest Thailand; LPC = Laos; MA: Vietnam; MKT: Southern Myanmar.

All the samples with successful SNP calls were used to evaluate the structure of each parasite population. After the SNP data were filtered with the threshold of missing data <50%, 686 isolates, including 135 from this study and 551 from the published data (Takala-Harrison et al., 2015), along with 570 SNP data by using the same NimbleGen microarray were used for PCA. PCA illustrates the first PC on the horizontal axis and the second and third PC on the vertical axis, respectively (**Figure 6**). The samples are colored by geographical location stratified by country in Southeast Asia. PCA results indicate a clear distinction between the isolates. Parasites from Southern China are clustered and separated from the isolates from western Cambodia, northwest Thailand, southern Myanmar, Vietnam, and Bangladesh in Southeast Asia (Takala-Harrison et al., 2015) (**Figure 6**).

**Figure 7:**
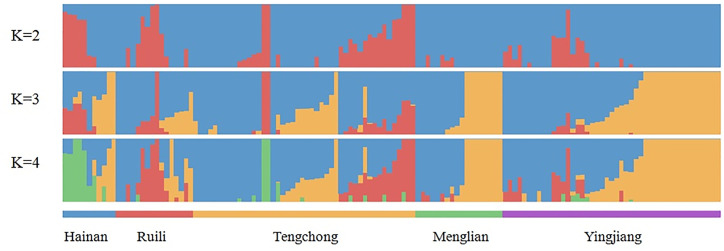
Population structure of the sample set, analyzed on the basis of geography, using ADMIXTURE. Parasites from the different sites in Yunnan Province along the border areas and Hainan Province were slightly separated.

The substructure of 135 samples of different origins in Southern China, including Yunnan and Hainan Provinces, was evaluated by ADMIXTURE. The parasite populations were designed on the basis of their geographic origins with K=5. Each vertical line represents a single sample with color denoting the origin proportion in that sample (**Figure 7**). Admixture estimates were computed using the parameters over a varying number of clusters (K) ranging from 2 to 10 with 10 technical replicates each. The optimal K value was determined by maximizing the log-likelihood across replicates for a single K value and minimizing the cross-validation error between different K values (**Supplementary File 2**). In this study, the optimal K was determined as 2, which indicates that a total of 135 samples from Southern China were mainly divided into two subgroups. The parasites from the different sites in Yunnan Province bordering with Myanmar belong to one group, and the parasites from Hainan Province are slightly separated from parasites from Yunnan Province (**Figure 7**).

## Discussion

Malaria was one of the most serious infectious diseases in the last century and was endemic in central and Southern China (Tang et al., 1997). However, China has made great contributions toward global malaria control in the past 40 years, and in 2010, China launched the National Malaria Elimination Action Plan 2010–2020 with an ultimate goal to interrupt local malaria transmission by 2020 (Feng et al., 2016; Yang and Zhou, 2016; Feng et al., 2020). ACTs, considered to be the best therapy for falciparum malaria in the world, have contributed to significant decreases in case numbers and deaths and are crucial to the success of control and elimination programs (Nosten and White, 2007; Dondorp et al., 2009). Recently, the emergence and spread of artemisinin resistance in *P. falciparum* poses a threat to malaria control and eradication goals in the GMS, where the resistance has emerged independently and has spread (Henriques et al., 2013; Ashley et al., 2014; Huang et al., 2015; Takala-Harrison et al., 2015; Ménard et al., 2016). Parasites in Southern China are reported to show decreased *in vitro* sensitivity to artemisinin and delayed parasite clearance time of artemisinin (Huang et al., 2015; Wang et al., 2015).

In this study, parasites collected from Southern China were genotyped using an SNP microarray, including 33,716 loci within the *P. falciparum* genome. This new SNP microarray was developed by using DBS, which did not have a sufficiently high concentration of DNA for sequencing (Jacob et al., 2014). We chose 64 samples with original DNA content of more than 2 ng and 71 samples of post-WGA DNA >2 ng. The average SNP call rate was 44.4% using the DBS in this study, and this was not as high as for venous blood samples (Jacob et al., 2014). WGA is one way to increase the total quantity of whole DNA, including human and parasite DNA (Nakayama et al., 2008; Chueh et al., 2011). Interestingly, the DNA concentration post-WGA was not correlated with the original DNA concentration in this study. The ratio of human DNA to parasite DNA prior to amplification may contribute to this discrepancy as a majority of the DNA sequences were from humans. Additionally, we observed a significant relation between the level of parasitemia and DNA quantity.

A total of 27 validated and candidate SNPs associated with antimalarial drug resistance were tested. Parasites in Southern China show a moderate prevalence of *pfcrt* mutation with a prevalence of 39.3%, which is much lower than that in another study that reported 100.0% mutant haplotype of *pfcrt* CIETS (Bai et al., 2018). However, the prevalence of *pfcrt* at Q271E, N326S, and I356L remain high in this study. Interestingly, the mutations of *pfmdr1* involving codons N86Y, Y184F, S1034C, N1042D, and D1246Y were all of wild type. CQ was used to treat falciparum malaria in China in the last century until it was withdrawn and replaced in the 1970s as a result of the CQ resistance emerging in Southern China according to *in vivo* and *in vitro* testing (Huang et al., 1988; Hong-Ping et al., 1998; Liu et al., 2005). This decreased prevalence of the *pfcrt* K76T marker may be associated with the cessation of CQ use against *P. falciparum* malaria in Southern China (Wang et al., 2005; Laufer et al., 2010).

The prevalence of *pfdhfr* and *pfdhps* remains at a high level even though antifolate drugs have not been used in China for many years, which is consistent with other studies (Huang et al., 2012a; Huang et al., 2012b; Bai et al., 2018). Pyrimethamine was used for the radical treatment of *P. vivax* in combination with primaquine in China around 40 years ago (unpublished data). Additionally, pyrimethamine plus primaquine has always been recommended as prophylaxis for specific populations, and pyrimethamine was added to salt for prophylaxis in China in the 1980s (Wang, 1984). The mechanism of action of *pfdhfr* and *pfdhps* mutations on the resistance to SP drugs has not been well described.

When the SNP microarray was developed, the K13 gene associated with artemisinin resistance was not identified. Therefore, only four candidate SNPs associated with delayed parasite clearance time were included in the microarray. Only one locus, MAL13:1719976, showed 100.0% mutation in all the samples, and the other three had low levels of mutation or were wild type. These results have provided further evidence of the decreased sensitivity to artemisinin and delayed parasite clearance time of artemisinin in Southern China, which was identified by our other published study (Huang et al., 2015).

According to the analysis of population structure, the parasites from Southern China represent a clear distinct cluster and little connection with the parasites from other countries in the GMS, which is consistent with the findings of other studies (Wei et al., 2015; Zhu et al., 2016; Ye et al., 2019). Although the population diversity of *P. falciparum* is high in the GMS, the parasites from Southern China are relatively low diversity. Human migration may be another cause for parasite population evolution and genomic diversity. Yunnan Province has a long border with Myanmar, but the human population movement is relatively low because of geographical barriers and some regions having political issues (Wang et al., 2016; Xu and Liu, 2016; Zhou et al., 2016; Chen et al., 2018). Hainan Province, geographically separated from the Chinese mainland, provides a natural barrier to parasite migration and spread. The population structure analysis is a useful tool that allows targeting of populations with low migration and low diversity in the malaria elimination stage and tracking of the origin of imported parasites in the postelimination stage.

## Limitations

In this study, we used samples of DBS from the field that had low quantity genomic DNA; even though we used WGA to increase the DNA amount, the total DNA were still lower than venous blood samples. In addition, the SNP call rates of DBS samples were not as good as those of venous blood samples. The DNA microarray could only test known SNPs. We did not test the SNPs of K13 using this microarray because K13 was not identified when the chip was developed. Although this custom array is not available now, some new microarrays have been developed, for example, Illumina Bead Chip microarray, protein array, or peptide array, which provide a high throughout and a powerful platform in malaria field (Bailey et al., 2020; Zhao et al., 2020).

## Conclusions

Parasites from Southern China were clustered and separated from those from Southeast Asia although parasites from Yunnan Province were a substructure of those from Hainan Province. This study provides a population-level genetic framework for investigating the biological origins of antimalarial drug resistance.

## Data Availability Statement

The raw data supporting the conclusions of this article will be made available by the authors, without undue reservation. Data from the JID paper that was used for comparison with the China samples is publicly available at: NIH Gene Expression Omnibus (www.ncbi.nlm.gov/geo/) (Accession number: GSE100704).

## Ethics Statement

The studies involving human participants were reviewed and approved by the National Institute of Parasitic Diseases, Chinese Center for Disease Control. Written informed consent to participate in this study was provided by the participants’ legal guardian/next of kin.

## Author Contributions

FH, CJ, and CP conceived and designed the study. CJ, ST-H, and FH carried out the data analysis. CJ, MA, and FH conducted the laboratory work. H-LY and HL collected the field samples. Z-GX, S-SZ, and L-HT contributed to the manuscript edits. FH drafted the manuscript. All authors contributed to the article and approved the submitted version.

## Funding

The study was supported by the Natural Science Foundation of Shanghai (No. 18ZR1443400), WHO Mekong Malaria Programme (WP/08/MVP/000512 and WP/10/MVP/005837), and the Howard Hughes Medical Institute and fellowship supported by Shanghai government. R01 (grant number R01AI101713) from the National Institute of Allergy and Infectious Diseases at the National Institutes of Health paid for the genotyping of the samples from the JID paper.

## Conflict of Interest

The authors declare that the research was conducted in the absence of any commercial or financial relationships that could be construed as a potential conflict of interest.
