# Supplementary material for: Genomic Epidemiology of Antimalarial Drug Resistance in Plasmodium falciparum in Southern China
Source: Front Cell Infect Microbiol. 2021 Jan 8;10:610985. doi: 10.3389/fcimb.2020.610985 (PMC7820777; doi:10.3389/fcimb.2020.610985)
Supplement: Supplementary file 1 [file DataSheet_1.docx]

**Supplementary file 1: Loci of antimalarial drug resistance markers**

| Codons | SNp | A | T | C | G | All |
| --- | --- | --- | --- | --- | --- | --- |
| *pfdhfr* 16 | MAL4:755115 | 0 | 0 | 2 | 51 | 53 |
| *pfdhfr* 51 | MAL4:755220 | 1 | 4 | 0 | 20 | 25 |
| *pfdhfr* 59 | MAL4:755243 | 0 | 0 | 16 | 24 | 40 |
| *pfdhfr* 108 | MAL4:755391 | 5 | 0 | 24 | 1 | 30 |
| *pfdhfr* 164 | MAL4:755558 | 0 | 3 | 0 | 45 | 48 |
| *pfmdr1* 86 | MAL5:958140 | 6 | 0 | 0 | 44 | 50 |
| *pfmdr1* 184 | MAL5:958435 | 3 | 0 | 3 | 7 | 13 |
| *pfmdr1* 1034 | MAL5:960984 | 17 | 0 | 0 | 28 | 45 |
| *pfmdr1* 1042 | MAL5:961008 | 39 | 0 | 1 | 0 | 40 |
| *pfmdr1* 1246 | MAL5:961620 | 0 | 0 | 3 | 44 | 47 |
| *pfcrt* 74 | MAL7:458998 | 0 | 0 | 0 | 66 | 66 |
| *pfcrt* 75 | MAL7:459001 | 0 | 0 | 0 | 51 | 51 |
| *pfcrt* 76 | MAL7:459003 | 1 | 0 | 11 | 16 | 28 |
| *pfcrt* 220 | MAL7:459785 | 0 | 3 | 5 | 23 | 31 |
| *pfcrt* 271 | MAL7:460214 | 1 | 0 | 17 | 24 | 42 |
| *pfcrt* 326 | MAL7:460740 | 0 | 0 | 3 | 31 | 34 |
| *pfcrt* 356 | MAL7:460978 | 0 | 0 | 66 | 0 | 66 |
| *pfcrt* 371 | MAL7:461216 | 0 | 2 | 0 | 56 | 58 |
| *pfdhps* 436 | MAL8:550802 | 0 | 1 | 0 | 64 | 65 |
| *pfdhps* 437 | MAL8:550806 | 0 | 0 | 7 | 9 | 16 |
| *pfdhps* 540 | MAL8:551114 | 0 | 0 | 20 | 25 | 45 |
| *pfdhps* 581 | MAL8:551238 | 0 | 0 | 18 | 42 | 60 |
| pfdhps 613 | MAL8:551333 | 0 | 0 | 0 | 55 | 55 |
| - | MAL10:688956 | 3 | 1 | 0 | 19 | 23 |
| - | MAL13:1718319 | 15 | 0 | 0 | 16 | 31 |
| - | MAL13:1719976 | 0 | 0 | 43 | 0 | 43 |
| - | MAL14:718269 | 0 | 0 | 9 | 30 | 39 |

Note: The numbers in yellow highlight are the samples of mutant gene at the different loci of antimalarial drug resistance markers.
