# Supplementary material for: Genomic Epidemiology of Antimalarial Drug Resistance in Plasmodium falciparum in Southern China
Source: Front Cell Infect Microbiol. 2021 Jan 8;10:610985. doi: 10.3389/fcimb.2020.610985 (PMC7820777; doi:10.3389/fcimb.2020.610985)

Supplementary file 2: Cross validation test result in ADMIXTURE analysis

The admixture software was used to analyze the population structure according to k = 2 to k = 10, 10 different seeds were selected for 10 repeated analysis, and then the results were clustered with Pong for 10 times, and the optimal K value was determined according to cross validation error. In this study, the optimal K is 2, which means that total 135 samples from Southern China are mainly divided into two subgroups.


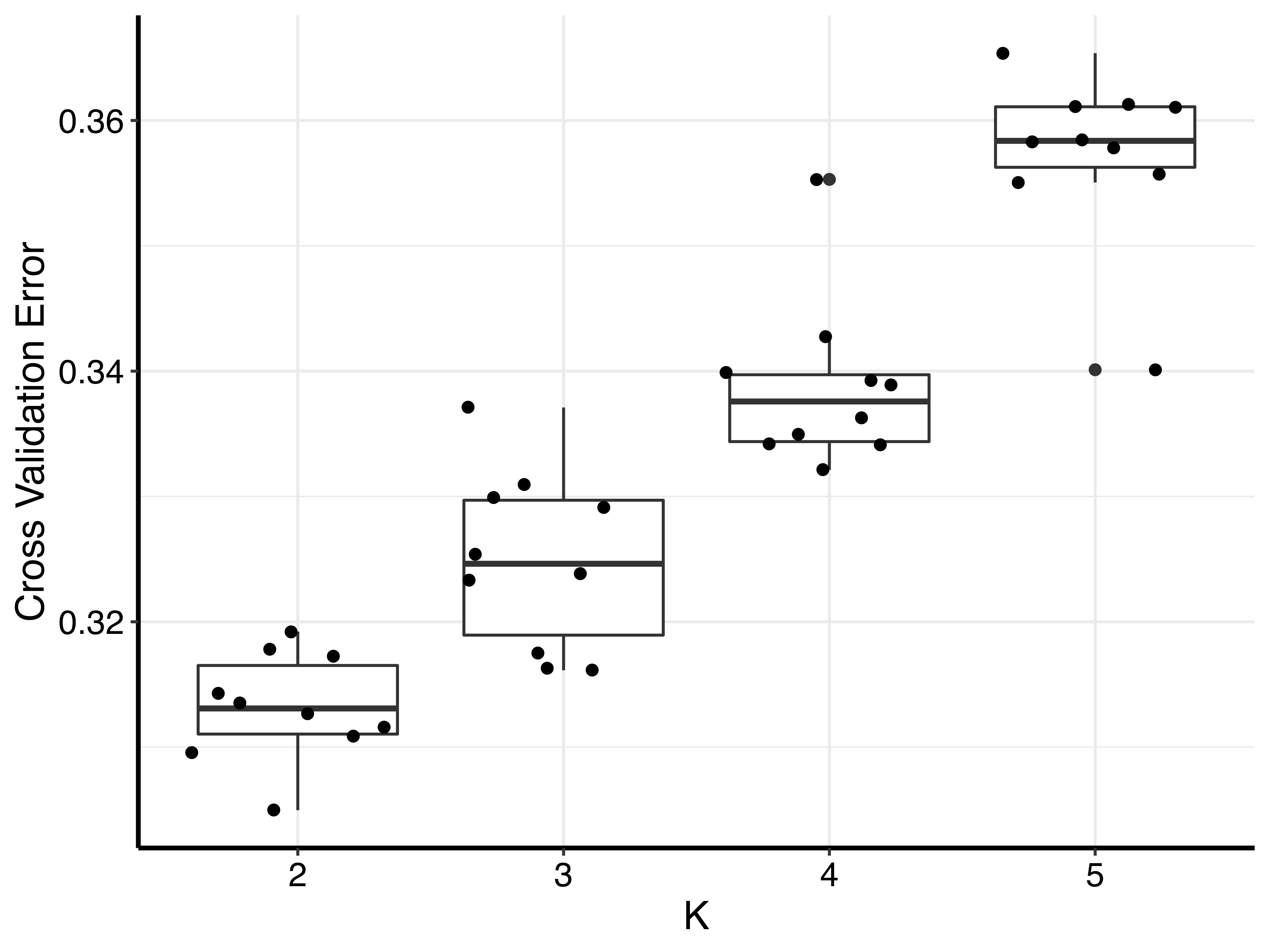

Supplement: Supplementary file 2 [file DataSheet_2.docx]
